# Identification of key genes and its chromosome regions linked to drought responses in leaves across different crops through meta-analysis of RNA-Seq data

**DOI:** 10.1186/s12870-019-1794-y

**Published:** 2019-05-10

**Authors:** Jubina Benny, Antonino Pisciotta, Tiziano Caruso, Federico Martinelli

**Affiliations:** Dipartimento di Biologia, Università degli Studi di Firenze, Via Madonna del Piano 6, Sesto Fiorentino, FI 50019 Italy

**Keywords:** Drought, Leaves, Meta-analysis, RNA-Seq, Seedlings, Transcriptomic

## Abstract

**Background:**

Our study is the first to provide RNA-Seq data analysis related to transcriptomic responses towards drought across different crops. The aim was to identify and map which genes play a key role in drought response on leaves across different crops. Forty-two RNA-seq samples were analyzed from 9 published studies in 7 plant species (*Arabidopsis thaliana*, *Solanum lycopersicum*, *Zea mays*, *Vitis vinifera*, *Malus X domestica*, *Solanum tuberosum*, *Triticum aestivum*).

**Results:**

Twenty-seven (16 up-regulated and 11 down-regulated) drought-regulated genes were commonly present in at least 7 of 9 studies, while 351 (147 up-regulated and 204 down-regulated) were commonly drought-regulated in 6 of 9 studies. Across all kind of leaves, the drought repressed gene-ontologies were related to the cell wall and membrane re-structuring such as wax biosynthesis, cell wall organization, fatty acid biosynthesis. On the other hand, drought-up-regulated biological processes were related to responses to osmotic stress, abscisic acid, water deprivation, abscisic-activated signalling pathway, salt stress, hydrogen peroxide treatment. A common metabolic feature linked to drought response in leaves is the repression of terpenoid pathways. There was an induction of AL1 (alfin-like), UGKYAH (trihelix), WRKY20, homeobox genes and members of the SET domain family in 6 of 9 studies. Several genes involved in detoxifying and antioxidant reactions, signalling pathways and cell protection were commonly modulated by drought across the 7 species. The chromosome (Chr) mapping of these key abiotic stress genes highlighted that Chr 4 in *Arabidopsis thaliana*, Chr 1 in *Zea mays*, Chr 2 and Chr 5 in *Triticum aestivum* contained a higher presence of drought-related genes compared to the other remaining chromosomes. In seedling studies, it is worth notice the up-regulation of ERF4 and ESE3 (ethylene), HVA22 (abscisic acid), TIR1 (auxin) and some transcription factors (MYB3, MYB94, MYB1, WRKY53 and WRKY20). In mature leaves, ERF1 and Alfin-like 1 were induced by drought while other transcription factors (YABBY5, ARR2, TRFL2) and genes involved phospholipid biosynthesis were repressed.

**Conclusions:**

The identified and mapped genes might be potential targets of molecular breeding activities to develop cultivars with enhanced drought resistance and tolerance across different crops.

**Electronic supplementary material:**

The online version of this article (10.1186/s12870-019-1794-y) contains supplementary material, which is available to authorized users.

## Background

Abiotic stresses are playing a major limiting factor affecting crop growth, yield and productivity [1, 2]. Among environmental stresses, drought is one of the most serious and increasing environmental factors affecting agricultural production. Drought stress affects water uptake and plant adaptation and long-term evolution of plant species to climate change [3]. To withstand in adverse environmental stress conditions like drought, the plant requires a substantial change in the metabolism, which includes regulation of transcription and gene expression and extensive transcriptome reprogramming upon the occurrence of the stress [4]. Therefore, transcriptomic studies offer great insight into the mechanisms of plant stress responses. Among the small plant molecules, hormones play an important role in the modulation of the complex plant physiological and molecular responses to drought. Abscisic acid is the key hormone modulating water loss and cellular growth maintenance [5]. However, this is only one among the many key players in the complex molecular networks underlying crop responses to environmental stresses. The outcome of the responses is regulated by complex crosstalk where small molecules (such as hormones) play a specific role of inhibition/induction of key proteins in stress signal reception, transmission and responses such as kinases, phosphatases, and transcription factors, defensive responsive genes [6]. Some key transcription factor (TF) families such as MYB, WRKY, bZIPs have been found to be involved in a different manner depending on the type of stress. Some TFs have been object of genetic engineering to improve stress tolerance in model and crop plants [7, 8]. Transcriptomic studies are essential in gaining insight into the crop responses to drought by identifying specific genes involved in plant responses to water stresses highlighting peculiarities of each crop and identifying which genes are the base of diverse drought tolerance and resistance mechanism. Since data of each study are typically related to only one season, this may lead to reduced reliability of the conclusions driven by each study. Indeed, it is essential to find a pipeline to compare data across species in order to strengthen the meaning of every single study, validating published works across species and reducing the environmental variability that affects their reliability. This kind of works, named meta-analysis is lacking in crops, especially at the transcriptomic level. Therefore, it is highly desirable to put more efforts in developing extensive studies to systematically understand drought-stress-related mechanisms in crops, which will accelerate the development of new crop varieties with improved stress resistance to increase agricultural sustainability and food supply for a highly growing world population.

RNA-Sequencing (RNA-Seq) is a rapid technique for genome-wide gene expression analysis [9–11]. With the emergence of this technique, the high-throughput transcriptomic technologies have been revolutionized. This technique can be considered as an efficient way to identify genes and gene families encoding proteins involved in different metabolic pathways related to the object of the study. Next-generation sequencing methods have enabled to understand the gene expression data in both quantitative and qualitative manner [12] and can be used for obtaining sequences on a large scale with high sequencing depth. To assist crop breeding in developing drought-tolerant crops, it is crucial to gain insight into the complex networks of crop environmental stress responses by elucidating the molecular basis of drought-stress transduction pathways and drought tolerance mechanisms. Omic approaches have been used to validate RNA-Seq data related to environmental stress responses [13, 14]. However, transcriptomic studies present some drawbacks represented by the following: 1) high presence of false-positive results that requires validation with other platforms, 2) data are generally affected by environmental, experimental, developmental and genetic conditions, 3) experiments are typically not repeated and conducted in only one season, 4) data are highly affected by the environment, especially when performed in field conditions, 5) few replicates are usually performed due to the high costs of these analysis and the scarce integration between transcriptomic and other omic platforms. Meta-analysis improve the reproducibility of RNA-Seq studies because: 1) it filters the most meaningful information linked with the object of study, 2) eliminates data affected by environmental variability, 3) reduces false positive results, 4) increase the number of virtual replicates, 5) integrates multiple datasets. Meta-analysis studies should be integrated with statistical modelling used for each sample in order to unveil intrinsic mechanisms [15]. In the present work, we have conducted a meta-analysis of 9 RNA-Seq studies conducted in 7 crops to deliver conserved and reliable genomic information exploitable by breeding to enhance drought resistance in crops. We analyzed (at a most comprehensive manner as possible) RNA-Seq data in crops (herbaceous, tree fruit crops, model plant) under drought using the same bioinformatics pipeline to deliver functional genomics knowledge that will guide molecular breeding to enhance drought tolerance and resistance in crops.

## Results

### Drought transcriptomic responses in all kind of leaves

Based on the search criteria described in Methods, we found 22 RNA-Seq studies: 7 were performed in roots, 12 in leaves, 3 in fruits. Among leaf studies, 3 of them have no raw data available. Indeed, the analysis was performed using 9 studies [16–24]: 5 dealing with mature leaves, 1 in young leaves and the other 3 in seedlings. The 9 studies comprise of 2 fruit tree crops and 5 herbaceous ones.

**Fig. 1:**
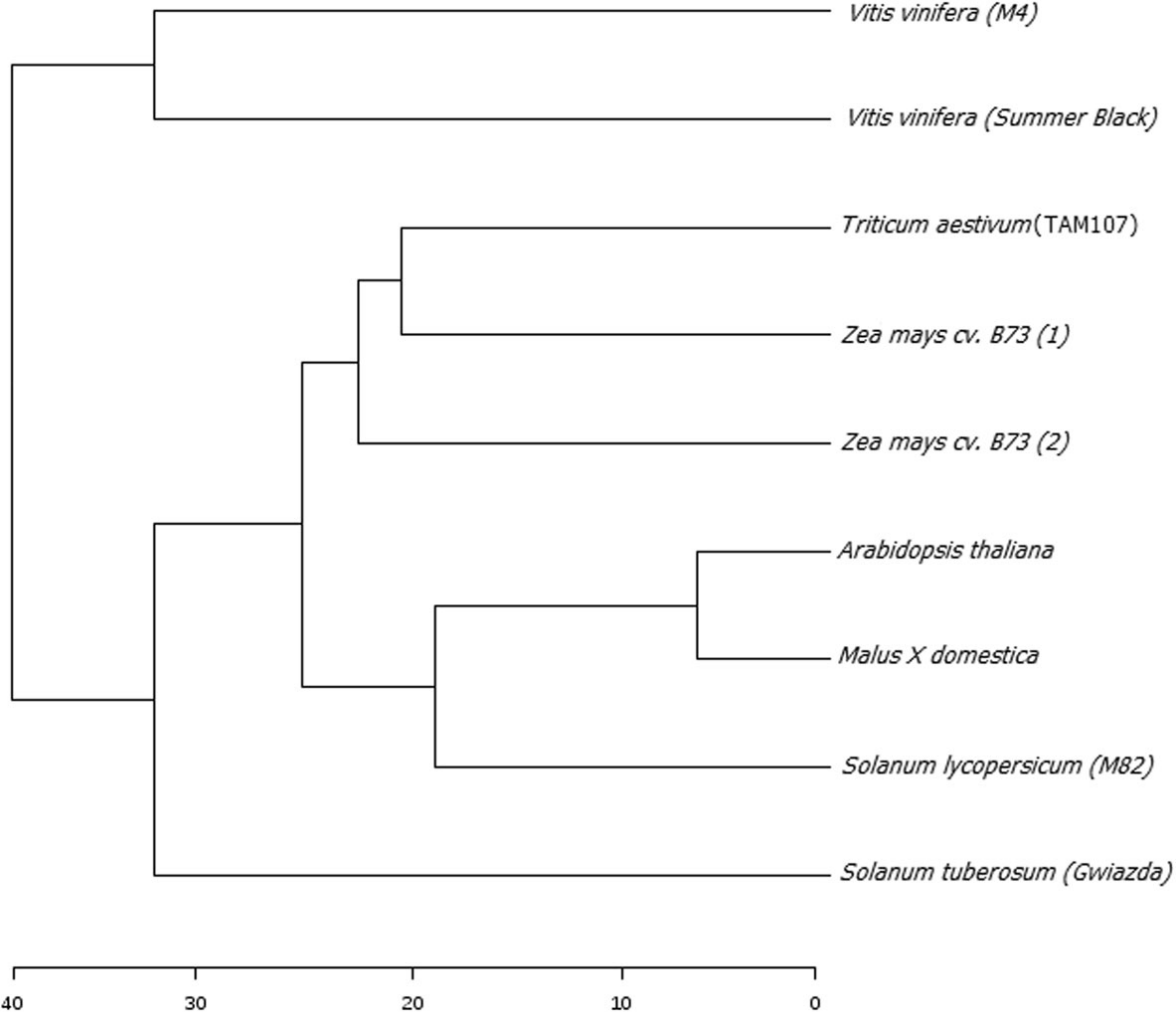
Dendrogram showing the hierarchical relationship among the RNA-seq studies selected for the meta-analysis. Resulted log2FC values of the analysis were used for generating the tree. Plant species used for the analysis (9 studies) were indicated

The articles and crops selected for the study, number of up- and down-regulated genes were listed in Table 1. The analysis resulted in the identification of a total of 108,903 genes in which 53,988 were up-regulated and 54,915 were down-regulated. For each of the analysis, the total number of genes range from 9420 to 17,230. The number of genes up-regulated was in a range of 2866 to 8184 and down-regulated genes were span from 4582 to 9046. The results of the analysis along the gene ID, *Arabidopsis thaliana* ortholog and log2 fold change value are provided in Additional file 1: Table S1. The two *Vitis vinifera* studies form a cluster showing an overall transcriptomic similarity towards the analysis to drought (Fig. 1; Additional file 2: Figure S1). Although the transcriptomic responses in the two maize studies were very similar, the closeness of one maize study to wheat study was higher than between the two maize studies. The similarity in drought responses among apple, *Arabidopsis thaliana* and tomato were related due to the fact that they dealt with drought responses in seedling leaves.

**Table 1:** The number of up-regulated and down-regulated genes in response to drought for each study. Number of up- and down-regulated genes in common in at least 6, 7, 8, 9 of 9 studies

Article	Crop	Sample Information		
		Total	Up	Down
Clauw et al. (2015)	*Arabidopsis thaliana*	17,230	8184	9046
Song et al. (2016)	*Zea mays cv. B73 (Study1)*	11,693	5611	6082
Corso et al. (2015)	*Vitis vinifera cv. M4*	11,114	6154	4960
Li et al. (2017)	*Zea Mays cv. B73 (Study 2)*	10,601	5225	5376
Pieczynski et al. (2018)	*Solanum tuberosum cv. Gwiazda*	10,843	6409	4434
Orcheski et al. (2016)	*Malus X domestica*	16,700	8545	8155
Liu et al. (2017)	*Solanum lycopersicum cv. M82*	9746	5164	4582
Haider et al. (2017)	*Vitis vinifera cv. Summer Black*	9420	2866	6554
Liu et al. (2015)	*Triticum aestivum cv. TAM107*	11,556	5830	5726
Commonly regulated in 9 of 9 articles		0	0	0
Commonly regulated in strictly 8 of 9 articles		12	5	7
Commonly regulated in strictly 7 of 9 articles		15	11	4
Commonly regulated in strictly 6 of 9 articles		351	147	204

### Gene set and pathway enrichment analysis

DAVID software was used to identify the common biological processes affected by drought at transcriptomic level considering the drought-regulated genes in at least 6 of 9 studies. Metabolic pathways divided in up- or down-regulated by drought along with GO ID, its GO term, count, *p*-values and Benjamini values were shown (Table 2). No GO-terms related to the biological process were commonly drought-regulated in at least 7 of 9 studies. Among at least 6 of 9 articles, 38 GO-terms were down-regulated while 28 were up-regulated. Among them, it is worthy to mention some of the biological pathways that are known to be repressed by the drought stress such as wax biosynthesis and cell wall organization, fatty acid biosynthesis, protein phosphorylation. On the opposite, we identified some GO-terms that were up-regulated in response to water stress such as response to osmotic stress, response to abscisic acid, response to water deprivation, abscisic-activated signalling pathway, response to salt stress, response to hydrogen peroxide.

**Table 2:** Significantly regulated biological processes (FDR < 0.05) which are commonly regulated in at least 6 of 9 transcriptomic studies

GO_ID	GO_TERM	Count	*P*-value	Benjamini test
DOWN-REGULATED				
GO:0006633	fatty acid biosynthetic process	3	7.02E-250	5.80E-247
GO:0015031	protein transport	6	2.93E-129	1.21E-126
GO:0016192	vesicle-mediated transport	4	1.08E-117	2.96E-115
GO:0046777	protein autophosphorylation	5	3.73E-117	7.70E-115
GO:0006468	protein phosphorylation	13	4.54E-32	7.49E-30
GO:0006839	mitochondrial transport	3	8.95E-26	1.23E-23
GO:0006096	glycolytic process	3	1.83E-23	2.16E-21
GO:0006412	Translation	9	7.14E-23	7.38E-21
GO:0000398	mRNA splicing, via spliceosome	3	3.95E-21	3.62E-19
GO:0071555	cell wall organization	3	3.95E-21	3.62E-19
GO:0006349	regulation of gene expression by genetic imprinting	3	4.28E-21	3.54E-19
GO:0010025	wax biosynthetic process	3	5.84E-21	4.39E-19
GO:0009611	response to wounding	5	4.47E-19	3.08E-17
GO:0006855	drug transmembrane transport	3	1.07E-17	6.77E-16
GO:0009409	response to cold	6	7.87E-15	4.65E-13
GO:0009553	embryo sac development	3	2.10E-12	1.15E-10
GO:0048364	root development	4	2.84E-12	1.47E-10
GO:0006629	lipid metabolic process	4	5.96E-11	2.90E-09
GO:0009058	biosynthetic process	3	3.39E-09	1.56E-07
GO:0009416	response to light stimulus	4	3.48E-08	1.51E-06
GO:0016310	Phosphorylation	4	4.45E-08	1.84E-06
GO:0009826	unidimensional cell growth	3	5.57E-08	2.19E-06
GO:0009723	response to ethylene	3	7.02E-08	2.64E-06
GO:0009751	response to salicylic acid	3	7.02E-08	2.64E-06
GO:0006508	Proteolysis	6	1.36E-07	4.89E-06
GO:0009555	pollen development	3	2.58E-07	8.86E-06
GO:0006886	intracellular protein transport	3	3.09E-07	1.02E-05
GO:0032259	Methylation	3	3.09E-07	1.02E-05
GO:0055085	transmembrane transport	3	3.09E-07	1.02E-05
GO:0045893	positive regulation of transcription, DNA-templated	3	4.61E-07	1.47E-05
GO:0051301	cell division	3	7.84E-07	2.40E-05
GO:0006511	ubiquitin-dependent protein catabolic process	3	7.84E-07	2.40E-05
GO:0006952	defense response	6	9.34E-07	2.76E-05
GO:0006457	protein folding	3	2.42E-06	6.90E-05
GO:0006979	response to oxidative stress	3	2.42E-06	6.90E-05
GO:0008152	metabolic process	3	3.55E-06	9.78E-05
GO:0007275	multicellular organism development	3	4.73E-06	1.26E-04
GO:0016567	protein ubiquitination	3	5.79E-06	1.49E-04
UP-REGULATED				
GO:0006351	transcription, DNA-templated	14	7.89E-06	1.98E-04
GO:0006355	regulation of transcription, DNA-templated	15	8.21E-06	1.99E-04
GO:0055114	oxidation-reduction process	6	1.23E-05	2.90E-04
GO:0006970	response to osmotic stress	5	3.37E-05	7.74E-04
GO:0009737	response to abscisic acid	8	3.64E-05	8.11E-04
GO:0009845	seed germination	4	3.82E-05	8.29E-04
GO:0006396	RNA processing	4	3.84E-05	8.13E-04
GO:0042542	response to hydrogen peroxide	3	5.36E-05	0.001106
GO:0009636	response to toxic substance	3	5.78E-05	0.001163
GO:0009408	response to heat	4	5.78E-05	0.001163
GO:0009624	response to nematode	3	6.12E-05	0.001203
GO:0009414	response to water deprivation	5	6.12E-05	0.001203
GO:0009734	auxin-activated signaling pathway	4	6.53E-05	0.001253
GO:0009738	abscisic acid-activated signaling pathway	4	7.53E-05	0.001412
GO:0009908	flower development	4	1.07E-04	0.001968
GO:0006470	protein dephosphorylation	3	1.29E-04	0.002319
GO:0006810	Transport	5	1.29E-04	0.002319
GO:0009651	response to salt stress	6	1.36E-04	0.00238
GO:0015979	Photosynthesis	3	1.36E-04	0.00238
GO:0009733	response to auxin	4	1.38E-04	0.002364
GO:0007165	signal transduction	5	1.51E-04	0.00255
GO:0009873	ethylene-activated signaling pathway	3	1.90E-04	0.003131
GO:0009735	response to cytokinin	3	2.35E-04	0.003805
GO:0035556	intracellular signal transduction	3	2.61E-04	0.004137
GO:0042742	defense response to bacterium	3	2.61E-04	0.004137
GO:0046686	response to cadmium ion	3	3.34E-04	0.005191
GO:0005975	carbohydrate metabolic process	3	3.35E-04	0.005117
GO:0009793	embryo development ending in seed dormancy	3	3.35E-04	0.005117

### Abiotic stress responses

**Fig. 2:**
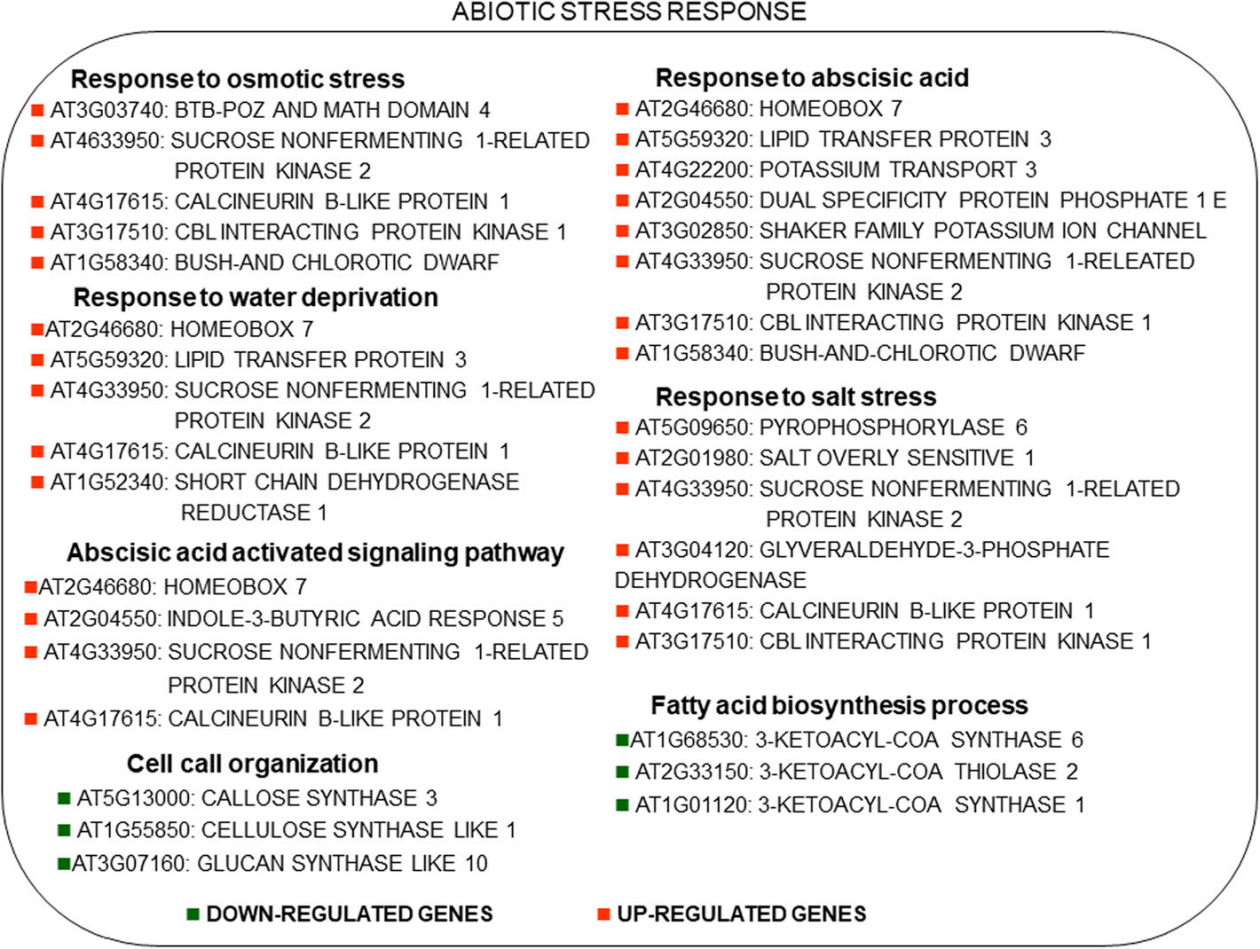
Drought-regulated genes involved in abiotic stress-related categories which are commonly regulated in at least 6 of 9 studies were indicated. Genes were identified as *Arabidopsis thaliana* orthologs of each gene of the analyzed plant species. Red indicated up-regulation and green indicated the down-regulation in response to drought

Genes mapped in the abiotic stress-related GO-terms identified by DAVID were shown in Fig. 2. Among the drought up-regulated genes involved in osmotic and salt stress, it is worth to mention the sucrose-related protein kinase and the CBL interacting protein kinase, the salt overly sensitive 1, and pyrophosphorylase 6. In the category of “response to water deprivation”, there was up-regulation of homeobox 7, lipid transfer protein 3, open stomata 1, calcineurin B-like protein. Four genes were up-regulated by drought and involved in “abscisic acid-activated signalling” while 8 of the drought up-regulated genes were involved in “response to abscisic acid”. Drought repressed three genes involved in fatty acid biosynthesis such as 3-ketoacyl-coa synthase 1 and 6 and 3-ketoacyl-coa thiolase 2.

### Secondary metabolism, cellular responses, signalling

MapMan web-tool was used to identify transcriptomic effects of drought in key selected categories such as secondary metabolism, cellular responses and signalling (Additional file 3: Figure S2). Among the secondary metabolism, the drought-repressed genes were involved in terpene pathways such as terpene cyclase, phytoene synthase, farnesyl pyrophosphate synthase 2. Cellular response genes were mostly inhibited by drought. MADS transcription factors like AGL8 (agamous-like MADS-box) and SCL3 (scarecrow-like protein) were enhanced. Relating to signalling mechanisms, genes encoding for 2 leucin rich repeat genes, 3 protein kinases, a proline-rich extension like receptor kinase, a lectin protein were repressed. On the other hand, a protein kinase (AT5G56890), and two serine/threonine kinases were up-regulated.

### Transcription factors and hormones

**Fig. 3:**
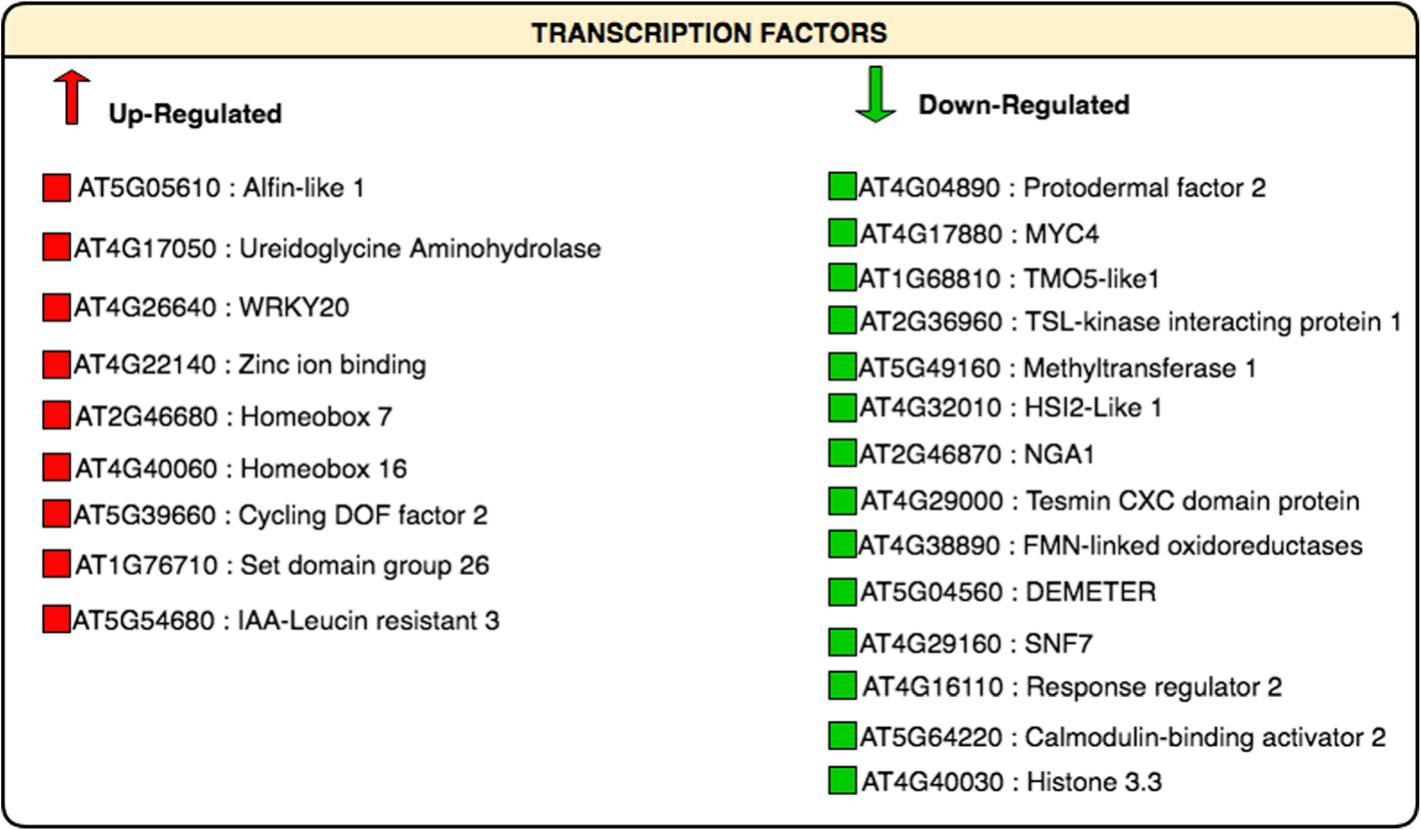
Drought-regulated genes involved in transcription factors which are commonly regulated in at least 6 of 9 studies. Genes were identified as *Arabidopsis thaliana* orthologs of each gene of the analyzed plant species. Red indicated up-regulation and green indicated the down-regulation in response to drought

**Fig. 4:**
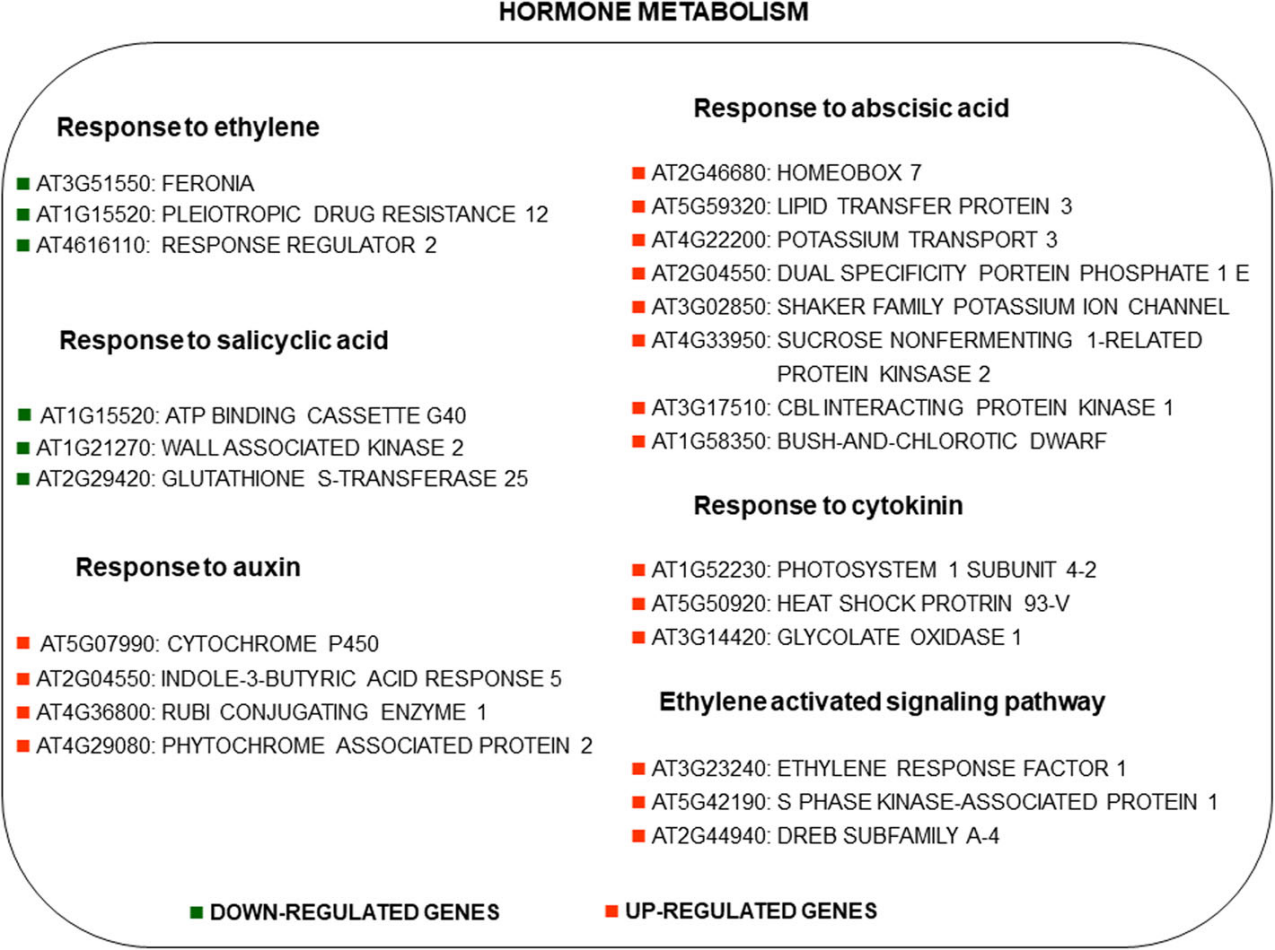
Drought-regulated genes involved in hormone-related categories which are commonly regulated in at least 6 of 9 studies were shown. Genes were identified as Arabidopsis orthologs of each gene of the analyzed plant species. Red indicated up-regulation and green indicated the down-regulation in response to drought

Among the drought-up regulated transcription factors, it is worthy to mention the induction of AL1 (alfin-like), UGKYAH (trihelix), WRKY20, zinc ion binding, two homeobox genes (one CDF2 and an SDG26 (SET domain)) (Fig. 3). Among the repressed ones, there were two bHLH members, a MYB factor (TKI1), two ABA-related TF (ABI3VP1), and ARR2 (cytokinin-related). Figure 4 summarized the drought-regulated genes involved in hormone-related categories. Ethylene and salicylic acid pathways were repressed by drought whereas auxin, abscisic acid, cytokinin, ethylene pathways were mostly up-regulated. Water deprivation down-regulated three genes responsive to ethylene and three responsive to salicylic acid (such as glutathione-s-transferase 2) while it up-regulated several genes responsive to auxin, abscisic acid, cytokinin and ethylene activated signalling pathway. Among the auxin-responsive genes it is worth to mention the enhancement of indole-3-butyric acid response 5 and the phytochrome associated protein 2. Relating to abscisic acid there was an up-regulation in homeobox 7, lipid transfer protein 3, shaker potassium ion channel, SNF1, potassium transport 3. The cytokinin responsive gene, heat shock protein 93, and three ethylene-related genes, ERF1, SKP1 and DREB were enhanced.

### Protein-protein network analysis

**Fig. 5:**
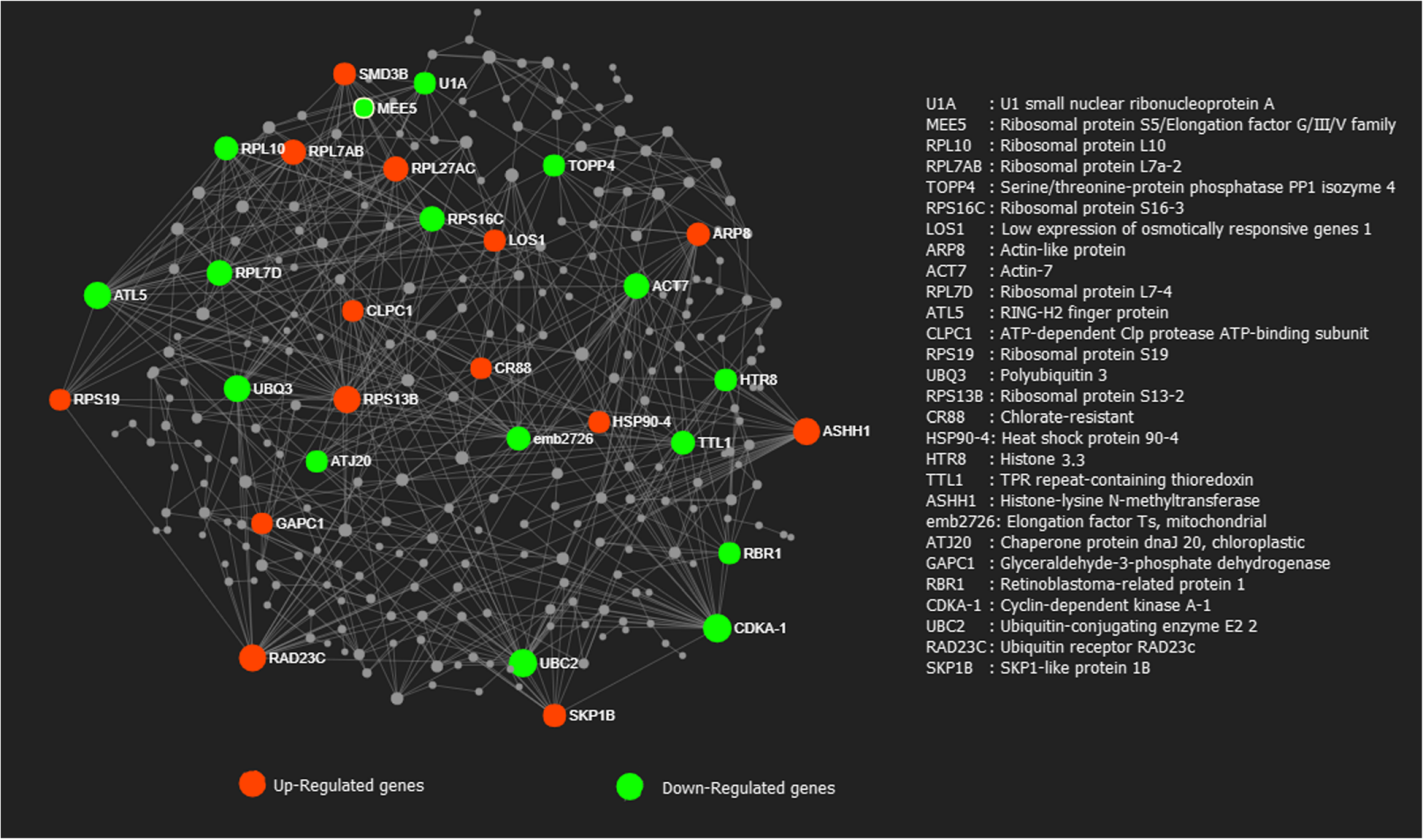
Protein-protein interaction network analysis predicted for genes commonly regulated in 6 of 9 studies based on Arabidopsis knowledgebase. Proteins encoded by genes having high degree of betweeness are shown in red color (up-regulated) and green color (down-regulated)

The protein-protein interaction (PPI) network analysis comprises of 351 drought-related genes commonly regulated in at least 6 of 9 studies. Minimum default settings were used to reduce the number of interacting proteins and the complexity of the networks (Fig. 5). Some key genes with a high number of interactions (> 20) were highlighted. Among the up-regulated hub (highly interacting) proteins it is worthy to notice some key proteins that may play a key role in drought response such as LOS1 (Low expression of osmotically responsive genes 1), HSP90–4 (heat shock protein 90–4), SKP1B (SKP1-like protein 1B), CR88 (chlorate resistant). Interestingly, drought down-regulated highly interactive proteins such as ATL5 (ring H2 finger protein), UBQ3 (polyubiquitin 3), TTL1 (TP repeat-containing thioredoxin), ATJ20, (chaperone protein DNAJ 20), CDKA-1 (Cyclin Dependent Kinase A-1). PPI network analysis was performed for drought-regulated in common between the three seedling studies and between the five studies on mature leaves (Additional file 4: Figure S3, Additional file 5: Figure S4). In seedling, two major hub proteins WDR5A (histone methylase component) and ASHH1 (histone lysine N methyltransferase) were up-regulated (Additional file 4: Figure S3). In mature leaves, an LRR receptor-like serine/threonine protein kinase was repressed while CDKF-1 (cyclin dependent kinase F-1) was up-regulated (Additional file 5: Figure S4).

### Chromosome mapping of key drought-regulated genes in crops

Key genes encoding genes related to abiotic stress responses, transcription factors, hormone metabolism (obtained from DAVID software) (respectively visualized in Figs. 2, 3, and 4) were mapped in the respective chromosomes of the 7 crops (Additional file 6: Figure S5; Additional file 7: Table S2). There were a total of 55 genes. Interestingly, we observed that in some species there was not a homogeneous distribution of these genes across chromosomes since some chromosomes contained a higher number of them. While in apple, potato and tomato there was a similar distribution of these genes in the chromosomes, whereas, in Zea mays, Triticum aestivum and *Arabidopsis thaliana* there was a higher presence of these genes in some of the chromosomes. In maize, a total of 29 abiotic stress-related genes were mapped to chromosome 1 implying that the chromosome 1 regions should contain more genes involved in drought resistance than the other chromosome regions. In *Arabidopsis thaliana*, 17 genes were mapped to chromosome 4. In *Triticum aestivum* chromosome 2 (2A + 2B + 2C + 2D) and 5 (5A + 5B + 5C + 5D genome) mapped respectively 15 and 12 genes. This work allowed to identify which chromosome might contain more genes involved in drought resistance and will guide the identification of new molecular markers linked with drought resistance.

### The most common drought-regulated genes

The 27 genes that were drought-regulated in at least 7 of 9 studies were considered to be closely linked to drought responses. They were mapped in each plant genome (Additional file 8: Figure S6; Additional file 9: Table S3). Ethylene response factor 1, protein kinase 2B, WRKY20 and Alfin-like 1 were up-regulated in at least 8 of 9 studies. At the same time, there was an inhibition of ABA-responsive element binding protein 3 and Protein phosphatase 2A subunit A2 in at least 8 of 9 studies. Among these highly conserved genes, a zinc-finger, ocre domain-containing protein 1, Rho GTPase-activating gaco-like protein and serine carboxypeptidase-like 27 were up-regulated while SLY1 gene family was down-regulated (Additional file 9: Table S3).

### Drought-regulated transcriptomic responses at different leaf stage

**Fig. 6:**
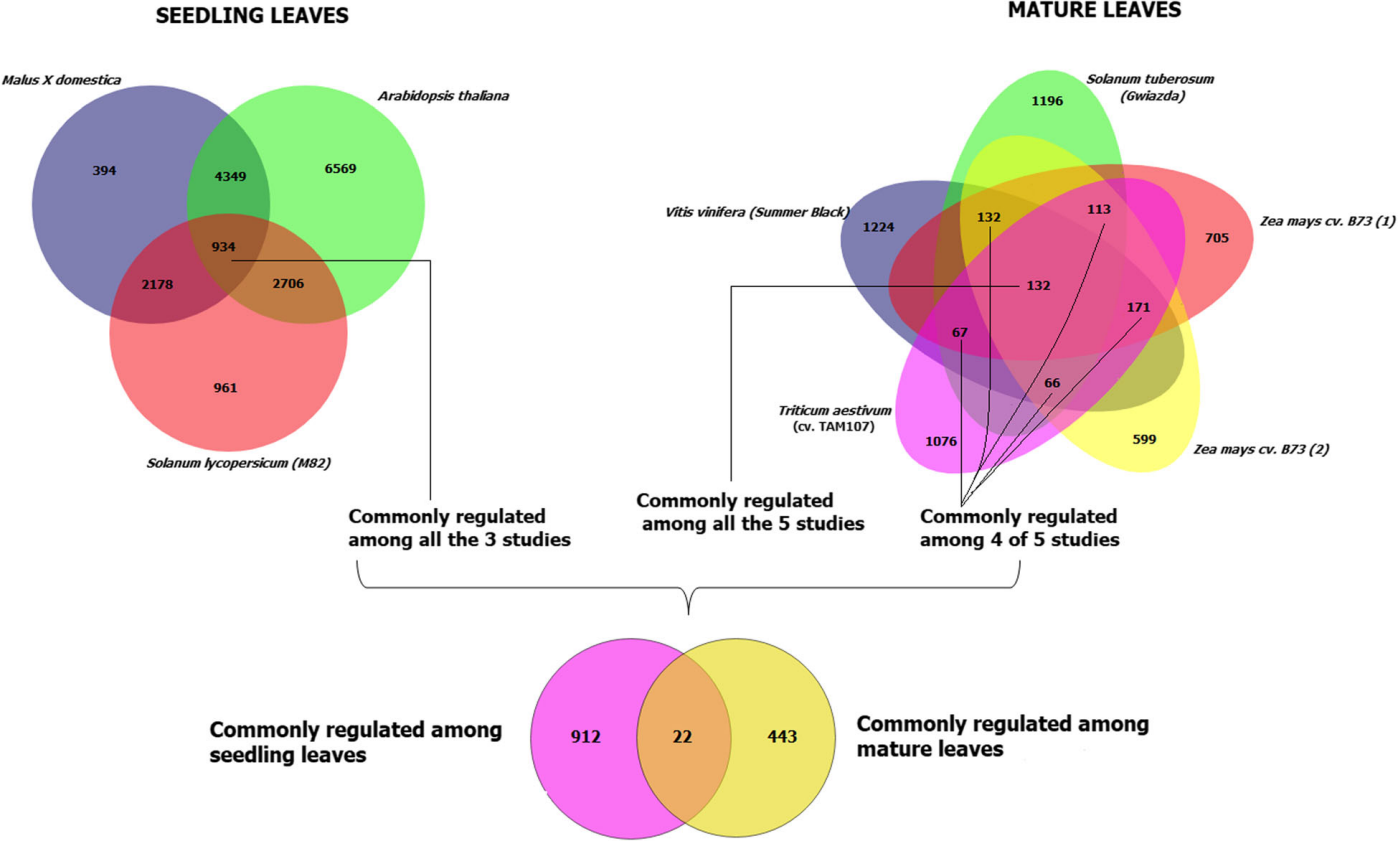
Comparison between transcriptomic responses to drought during leaf development (in seedlings and mature leaves). Venn-diagram showing the number of commonly regulated and unique genes responsive to drought in the three seedling studies and in the five studies dealing with mature leaves

Attention was paid on the drought-responsive genes at different leaf developmental stages. Comparing the three studies dealing with drought transcriptomic responses in seedlings (tomato, *Arabidopsis thaliana* and apple), 934 commonly drought-regulated genes were identified (Fig. 6). On the other hand, 465 genes were commonly drought-regulated in at least 4 of 5 studies in mature leaf tissues. Finally, comparing the two lists of drought-regulated genes, 912 genes were specifically drought-regulated in seedlings, 443 in mature leaves and 22 in common between the two types of leaves (Additional file 10: Table S4 and Additional file 12: Table S5). These results demonstrated that transcriptome reprogramming in response to drought depends on different leaf developmental stage.

### Drought-responsive genes in seedlings

Among the genes that were regulated by drought in seedlings, we paid our attention to those belonging to key categories playing an important role in drought response modulation such as hormones, transcription factors and abiotic defence responses (Additional file 10: Table S4). Relating to hormones, two ethylene-related genes (ERF4 and ethylene-responsive element binding protein (ESE3, AT5G25190)) were up-regulated in all the three seedling studies. In addition, there were other 5 up-regulated genes involved in auxin (TIR1, auxin-responsive protein (AT4G38840)), abscisic acid (HVA22), gibberellin (KAO2, GA4). On the other hand, there were 8 down-regulated hormone-related genes: oxidoreductase B2 and AIR9 (auxin-related), AREB3 (abscisic acid), BAK1 (brassinosteroid-related), CKX7(cytokinins), gibberellin-20-oxidase2 and gibberellin-2-beta-dioxygenase (gibberellins). Three genes involved thioredoxin pathways were also up-regulated: APRL5, PDIL5–1, ATY1. Unexpectedly there were also some heat stress-related genes repressed such as (HSP17.8, ARL1, GFA2, HSP98.7). Regarding with transcription factors, there were some key categories that were commonly up-regulated among crops such as MYBs (MYB3, MYB94, MYB1), bHLH, and homeobox. Relating to the WRKY family, two genes were up-regulated (WRKY53 and WRKY20) while one gene was repressed (WRKY22). Interestingly, the SET-domain family was mainly up-regulated.

### Drought-responsive genes in mature leaves

We focused our attention on genes involved in the same categories that are commonly drought-regulated in at least 4 of 5 studies (cellular responses, hormones, transcription factors) (Additional file 10: Table S4). In total 4 hormone-related genes were drought-repressed such as an auxin-responsive (RRT4; O-fucosyltransferase family protein), two ABA-related genes (NCED4, HVA22A) and one salicylic acid-related (UDP-glucosyltransferase). ERF1, a key player in jasmonic acid-ethylene crosstalk was up-regulated by drought in mature leaves. Unexpectedly we observed that most of the genes encoding transcription factors were repressed by drought including YABBY5, ARR2, BLH6, TRFL2, three zinc finger proteins and other 5 genes. Alfin-like 1 was the only up-regulated transcription factor. Relating to another primary metabolism, it is worth notice that two genes involved in phospholipid biosynthesis were repressed (phosphatidylserine synthase and galactolipid galactosyltransferase).

## Discussion

Meta-analysis of transcriptomic responses to abiotic stresses has been performed comparing different studies that were conducted in the model plant *Arabidopsis thaliana* [25], rice [26], sunflower [27]. Our study used a similar approach expanding the analysis across different crop species. Our aim was to shed light into drought response mechanisms conserved across crops instead of identifying specific responses in each crop. Our purpose was mainly to answer the two following unresolved questions:

1. Which genes and molecular mechanisms are conserved across species and can be considered strictly modulating drought responses in plants?
2. How leaf development affects crop molecular responses to drought and which genes are playing a key role in drought resistance at different developmental stages?

### Common drought responses across plant species in all kind of leaves

We answered the first question by identifying drought-regulated genes in common across plant species. Twenty-seven genes were up- or down-regulated in response to drought in at least 7 of 9 studies. Some of them required particular attention considering that they have been previously linked to drought responses in single studies. They were ERF1 (involved in ethylene signalling), WRKY20 and Alfin-like 1, zinc finger ocre domain protein 1 (transcription factors), serine carboxypeptidase 27 and protein kinase 2B (involved in signalling). The involvement of these genes in drought responses is discussed below.

Among the drought-regulated 351 genes in at least 6 of 9 studies, our attention was paid on the hormone, transcription factor and stress defence categories. Among those genes related to osmotic stress, the up-regulation of sucrose nonfermenting1–related protein kinase2 (SNF1-related protein kinase; also named as SnRK2) was conserved across species. This member belongs to a family of genes that have been previously associated with osmotic stresses [28, 29]. [30] showed that these members are induced by osmotic stress and that three of them are activated through an ABA-dependent manner. Its role is extremely important in guard cells where it is playing a key role as a central hub to mediate ABA signalling [31]. Taken together, our meta-analysis confirmed that this gene should be an important player in sensing water deprivation in leaf tissues in different crops. This gene should be considered as a target for crop genetic engineering for the development of molecular markers associated with drought-resistance in crops.

Our work identified two genes involved in abiotic stress signalling: a calcineurin B-like (CBL) calcium sensor protein and a CBL interacting protein kinase 1. Calcineurin B-like proteins (CBLs) represent a unique family of plant calcium sensors that relay signals by interacting with a family of protein kinases, designated as CBL-interacting protein kinases (CIPKs). A previous study indicated that CIPK23 play this important role in water stress response by interaction with the calcium sensors CBL1 and CBL9 that synergistically regulates CIPK23. As suggested by [32], the different combination of CIPK and CBL members should be responsible for cell-specific signalling responses (osmotic stress or potassium uptake) in different organs (leaves or roots). Based on these findings, we can speculate that the simultaneous induction of calcineurin B-like calcium sensor protein (AT4G17615) and CBL interacting protein kinase 1 (AT3G17510) across 6 of 9 studies implies that the two proteins should play a major role in the activation of rapid drought sensing. This hypothesis implies that these two genes might be also considered as good targets for molecular breeding to enhance abiotic stress resistance.

Relating to GO term “response to water deprivation”, three additional genes were up-regulated such as homeobox 7, lipid transfer protein 3 (LTP3), short dehydrogenase reductase 1 (ABA2). Homeobox 7 belongs to Homeodomain-leucine zipper (HD-Zip) family proteins which are transcription factors related to environmental stress responses in plants. A member of homeobox family has been shown to confer resistance to drought in *Helianthus annuus* (sunflower) through over-expression [33]. LTP3 is known to bind to lipids and its over-expression enhanced drought tolerance through the action of MYB96 that directly binds to its promoter [34]. ABA2 is a NAD- or NADP-dependent oxidoreductases involved in ABA biosynthesis. This gene is responsive to ABA exogenous treatment [35]. Our analysis showed that a SOS3 like calcium binding protein was commonly induced by drought in 6 of 9 crops. This gene encodes a member of the calcineurin B-like calcium sensor gene family and mediates salt tolerance by regulating ion homeostasis in Arabidopsis. We also observed an up-regulation of salt over sensitive 1 (SOS1) that is a key player of the Salt-Overly-Sensitive (SOS) pathway, essential for maintaining a normal ion ratio in the cytoplasm in salt conditions [36]. Salinity is biphasic stress composed by an initial change of osmotic conditions followed by a subsequent stage of ionic modifications. Indeed, SOS1 plays an important role in the second phase of salinity stress. Transgenic over-expression of this gene has shown to induce drought tolerance in *Arabidopsis thaliana* demonstrating that improved resistance to salt stress can be obtained by limiting Na + accumulation in plants [37]. Being drought mainly osmotic stress, the induction of this gene implies a possible role of this gene in the response to osmotic changes too. This might be explained by the fact that water deprivation has the consequence to increase the levels of soil ion concentrations which indirectly causing salt stress. Our meta-analysis highlighted the repression of fatty acid biosynthesis in response to drought in leaves. It is known that water deficit inhibits fatty acid desaturation and drought resistance has been linked with a reduction of fatty acid metabolism in cotton resulting in greater stability of the membrane system [38].

Relating to hormones, we found several drought-regulated genes in common between 6 of 9 studies. Three genes were involved in ABA biosynthesis and signalling, two genes in auxin response, two genes involved in ethylene-related pathways. ABA2 *Arabidopsis thaliana* mutants showed a reduced drought tolerance in comparison to wild type [39] implying that the up-regulation of this gene should be a benefit for drought resistance. Our meta-analysis showed another unexpected result: ABA3 was repressed in response to water stress in 6 of 9 crops. This gene is a basic leucine zipper (bZIP)-type ABRE-binding protein that was shown to be up-regulated by drought in vegetative tissues [40]. Although at first glance, our results on both AREB2 and HVA22 seem to be in contrast with published findings, the repression of this gene in response to drought might be due to differences in the analyzed time points and drought intensity between studies.

Relating to ethylene biosynthesis, we found that ACS12 was constitutively repressed by drought. It is generally accepted that ethylene is involved in mediating plant responses to abiotic stress [41]. ACS cereal mutants showed to have delayed leaf senescence in drought conditions. Mutant leaves continue to be photosynthetically active under water stress implying that leaf function is maintained [41]. These findings showed that ethylene may serve to determine the onset of natural senescence and regulate drought-induced senescence. Based on these findings, we may speculate that the repression of ACS12 in leaves should be beneficial to inhibit ethylene biosynthesis and consequently improve drought resistance. ERF1 is known to be involved in plant disease resistance [42] but its role in abiotic stress responses is less clear. ERF proteins are characterized by an ERF DNA binding domain. These transcription factors bind to multiple cis-elements such as DRE/CRT and CE1 elements, involved in stress responses [43]. In *Arabidopsis thaliana*, the expression of ERF1 enhanced tolerance to drought. [44] hypothesized that ERF1 was linked with enhanced drought resistance in rice through the induction of ABA2. Since we found that both ABA2 and ERF1 were induced in 6 of 9 crops, our findings confirm this hypothesis rendering these two genes potential targets for enhancing resistance to drought. Among the conserved drought up-regulated transcription factors, it is worth to mention WRKY20, a member of WRKYs. This finding is in agreement with published works that demonstrated an increased drought tolerance due to the over-expression of WRKY20 in *Arabidopsis thaliana* [45].

### Drought-regulated genes at different leaf developmental stages

We answered the second question by identifying the drought-regulated genes commonly expressed among the three transcriptomic studies dealing with seedling responses and among the five studies performed on mature leaves.

Our findings highlighted that drought has very different transcriptomic effects on leaves depending on their developmental stage. Indeed, the identification of expression QTLs for drought resistance in leaves should clearly take in high consideration which developmental stage is considered. Among the 22 drought-regulated genes commonly expressed seedling and mature leaves it is worth noticing a transcription factor (alfin-like 1) and a heat shock protein (HSC70–7). Several members of Alfin-like TFs were up-regulated in response to different abiotic stresses in *Brassica oleracea*. The role Alfin-like TFs in enhancing salt stress and drought resistance is well-known when it is over-expressed in roots [46, 47]. Alfin-like 1 is a transcription co-activator [48] that contains a typical PHD finger binding promoter element of PRP2, a salt inducible gene [49]*.* Our meta-analysis lets hypothesize that the role of this transcription factor might have a similar function in leaves.

### Drought-regulated genes in seedlings

The up-regulation of two ethylene signalling genes in seedlings (ERF4 and one EREBP (ESE3)) implies that ethylene might have a promoting effect in drought response at early leaf development. These findings agree with previously published works that showed that the over-expression of ERF4 promoted adaptation to salt stress and drought [50]. This gene is a transcriptional repressor that suppressing a repressor of defence response genes positively regulates shoot growth and water-stress tolerance in rice during early growth stages [51]. ESE3 belongs to a family of ethylene response factor (ERF) genes that are involved in enhancing salt tolerance. Results of our work confirmed that the up-regulation of ethylene signalling should play a key role in drought resistance. HVA22 is an ABA-responsive gene regulated by environmental stresses. The up-regulation of HVA22 has been shown to be tissue-specific and in response to drought in barley [52]. Our analysis confirmed this evidence showing an opposite trend of expression between seedlings and mature leaves. Our results challenged the hypothesis that this gene should enhance drought resistance in mature leaves.

MYB is a large family of transcription factors well-known to be involved in drought. The transgenic over-expression of MYB1 enhanced drought resistance [53]. MYB94 activates cuticular wax biosynthesis in *Arabidopsis thaliana* and might be important in drought response [54]. Our analysis confirmed the role of these two MYB factors in seedling response to drought implying that they should be considered potential targets for enhancing drought resistance. The induction of MYB factors in drought is reported in the selected articles [16, 18, 19, 22]. We also found that WRKY53 was up-regulated in seedlings in response to drought confirming previous findings that showed WRKY53 drives the inhibition of stomatal closure by reducing H_2_O_2_ content facilitating stomatal opening by promoting starch degradation [55] and consequently inhibiting drought tolerance. The induction of WRKY20 in response to drought should allow a positive effects on drought tolerance in crops since the over-expression of this gene improved plant yields in soybean and enhanced drought tolerance in alfalfa [56]. Our protein-protein interaction analysis showed that WDR5A was up-regulated in all three seedling studies in response to drought. This confirmed the important role of this protein in drought responses. WDR5A is a regulating nitric oxide accumulation and NOS-like activity in guard cells to modulate stomatal closure for adaptive plant response to drought [57]. In seedlings, this gene should drive the closure of the stomata and the survival of leaf cells under water deprivation.

### Drought-regulated genes in mature leaves

In mature leaves, our meta-analysis found that ERF1 was up-regulated. The same result can be seen in [17]. The role of this gene in drought response has been previously discussed. Considering the mature leaf datasets all together, the role of ERF1 in modulating the expression of anti-oxidant and detoxifying proteins that protect cell components in leaf mature tissues is highlighted. ERF1 should work as a ‘regulatory gene’ under different stress conditions, changing the expression of ‘functional genes’ acting as detoxification and osmotic adjustment enzymes or proteins to protect cells from damage.

BAG6 is a Calmodulin (CaM)-binding transcription activators (CAMTA), which translates calcium signatures into different biochemical, and molecular pathways [58] and acts as a multi-functional protein that regulates apoptotic-like processes involved in different abiotic stresses. We found that this gene was up-regulated in mature leaves across the different crops. Indeed, we speculate that this gene should be involved in signalling mechanisms in response to drought stress. Calcium (Ca2+) works as a secondary messenger in plants and it is involved in different responses to different environmental stresses [59]. These transcription factors modulate many functional genes involved in stress tolerance in plants including drought [60] and regulate the expression of ERFs [61]. Based on these findings we may hypothesize that the two genes BAG6 and ERF1 might be linked in a common signalling response to drought in crops in mature leaves. Interestingly our analysis showed that HSP70 was repressed by drought. The heat shock protein 70s (Hsp70s) and heat shock factors (Hsfs) play key roles in protecting plant cells or tissues from various abiotic stresses [19]. It was observed that heat shock proteins play as activators or repressors, suggesting that these proteins might be modulated by both the activation and the repression mechanisms under stress condition [62]. Indeed, the effect of the repression of HSP70 in mature leaves under drought has to be further investigated. Cyclin dependent kinases (CDKs) are signalling proteins induced by stresses such as drought [63]. Since this gene was a highly interacting protein in drought-related gene networks, we may speculate that the induction of CDKF1 in mature leaves should play an important role in the promotion of drought resistance in crops.

## Conclusions

**Fig. 7:**
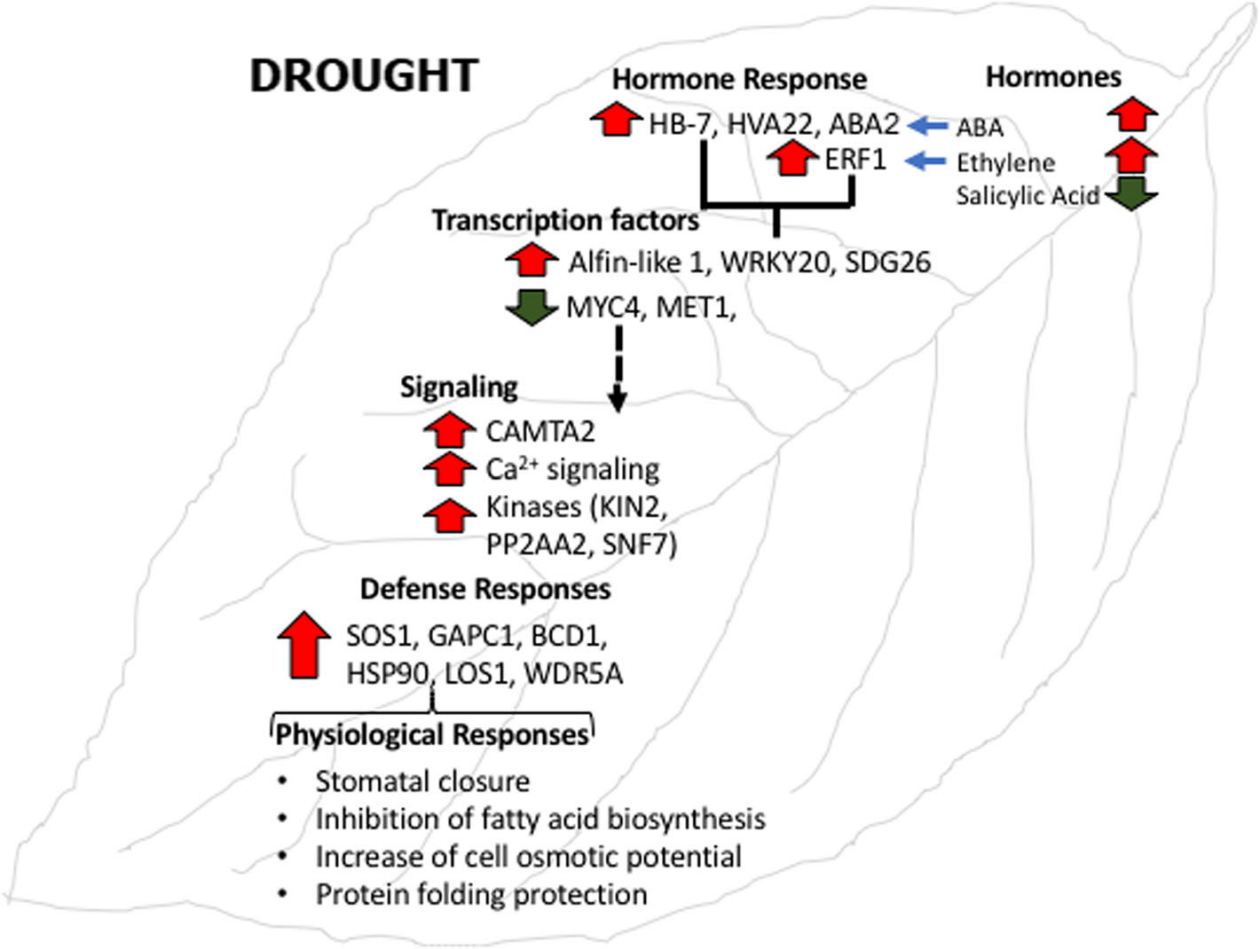
A model of transcriptional modulation of plant responses to drought in leaves. Important genes identified by the meta-analysis belonging to key functional categories and their consequent involvement in physiological responses were indicated

Taken together all these findings, we propose a model of plant response to drought shown in Fig. 7. The first plant response should be the induction of the biosynthesis of key hormones such as ABA and ethylene driving the activation of key signalling proteins (ERF1, ABA2 and HB7). These proteins should promote the fine-tuned transcriptional modulation through the cross-talk of a complex network of transcription factors (Alfin-like 1, WRKY20, SDG26). The up-regulation of key proteins in the signal transduction (CAMTA2, KIN2, and SNF7) should provoke the induction of proteins involved in physiological defensive responses represented by stomatal closure, inhibition of fatty acid biosynthesis, an increase of osmotic potential and protection of protein folding. Molecular breeding for drought resistance should focus on these genes.

This work allowed identifying genes involved in drought responses that were conserved across crop species. The mapping of genes in the different chromosomes of the 7 analyzed crops will help in developing new molecular markers for drought resistance. This gene mapping allowed identifying which chromosomes contain more DNA regions have to be taken in higher consideration for the breeding of crops in relation to drought resistance. Our approach will be the example of similar studies dealing with the identification of key genes involved in resistance to other limiting biotic and abiotic stresses in crops.

## Methods

### Search strategy for selection of RNA-Seq studies

**Fig. 8:**
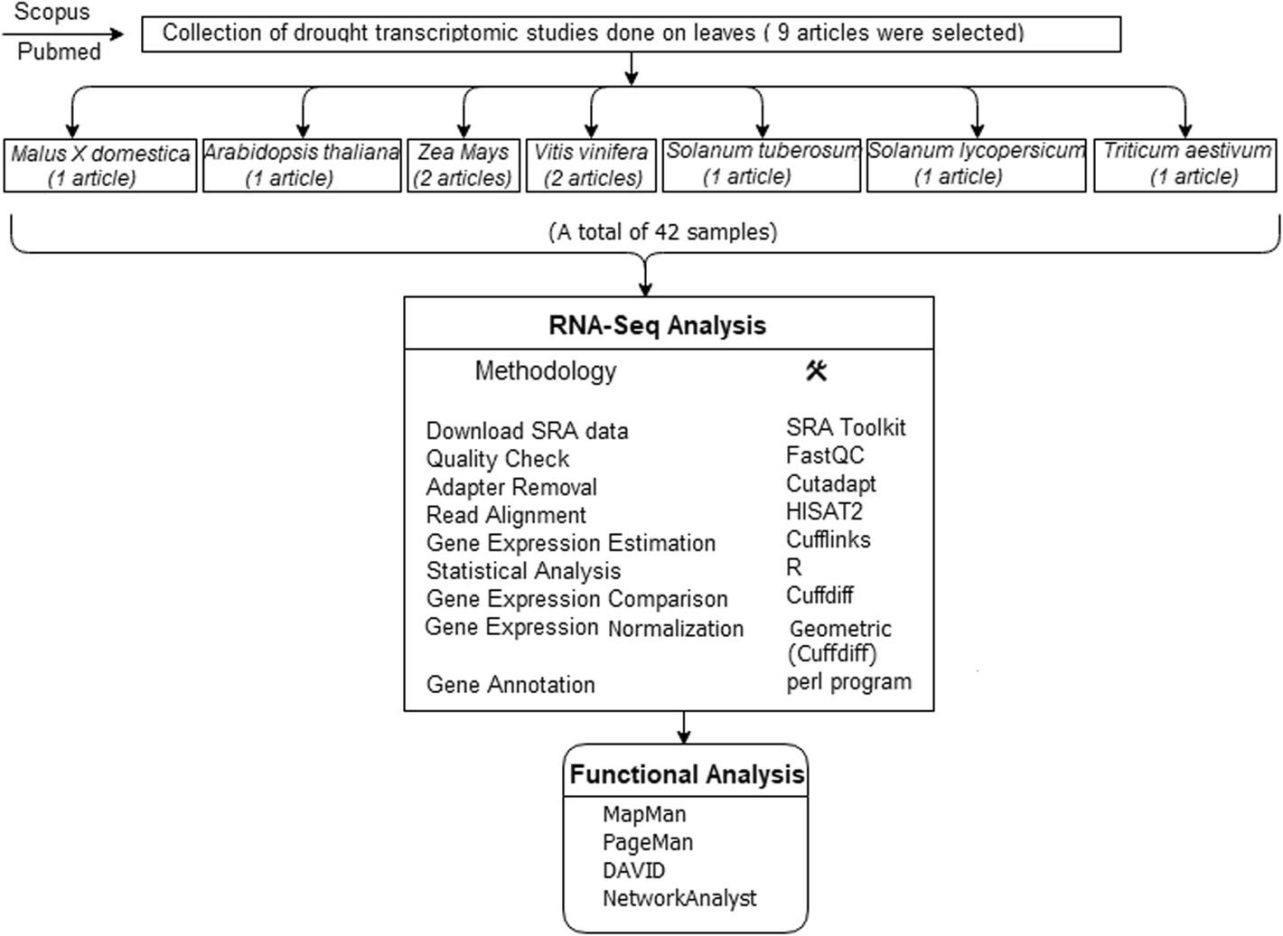
Workflow of the meta-analysis of the 9 transcriptomic studies related with drought stress in leaf tissue. Functional and statistical data analysis were indicated

Articles published dealing in response to drought in both tree fruit crop and herbaceous species were collected. These studies were identified from Scopus and PubMed if they respect the following criteria as follows: (i) consist of RNA-seq analysis, (ii) included at least one of the following terms in title and abstract: drought, leaf, stress, abiotic stress, water stress, (iii) studies provided raw data submitted in public databases. These criteria resulted in a selection of 9 articles comprising of total 42 samples (Table 3). If different time point series were present in the same study, a single time point was selected (10 DAS or whichever is nearer). This selection was done since most of the analyzed studies were performed at 10DAS. The selected studies were grouped based on leaf developmental stage (seedling leaves, young and mature leaves). Raw data were downloaded and analyzed through a pipeline previously used in similar meta-analysis [64]. The complete pipeline used for this study was provided in Fig. 8. The drought-modulated genes identified from each of the 9 studies were given in Additional file 11: Table S6.

**Table 3:** Articles, crops, number of samples, tissue and sample description (control vs treatment) included in the analysis

Articles	Crops	No. of Samples	Tissue	Sample Description		Duration of stress
				Control	Treated	
Clauw et al. (2015)	*Arabidopsis thaliana*	6	Seedling leaves	Control1 (ERR754071) Control2 (ERR754083) Control3 (ERR754090)	Treated1 (ERR754061) Treated1 (ERR754065) Treated3 (ERR754082)	The third seedling leaves were harvested at 10 DAS (Days after stress)
Song et al. (2016)	*Zea mays* *cv. B73 (Study1)*	2	Mature leaves	Control (SRR4054956)	Treated (SRR4048280)	Leaves were collected after 15 DAS
Corso et al. (2015)	*Vitis vinifera* *cv. M4*	4	Young leaves	Control1 (SAMN02393571) Control2 (SAMN02393572)	Treated1 (SAMN02393596) Treated2 (SAMN02393595)	Leaves were collected after 10 DAS
Li et al. (2017)	*Zea Mays* *cv. B73* *(Study 2)*	4	Mature leaves	Control1 (SRR3984708)Control2 (SRR3984749)	Drought1 (SRR3984782) Drought2 (SRR3984791)	Plant were grown without watering until their third leaves were fully expanded
Pieczynski et al. (2018)	*Solanum tuberosum* *cv. Gwiazda*	10	Mature leaves	Gwiazda_D0_1 (SRR5448182)Gwiazda_D0_2 (SRR5448183) Gwiazda_D0_3 (SRR5448184)	Gwiazda_D6_1 (SRR5448185) Gwiazda_D6_2 (SRR5448186) Gwiazda_D6_3 (SRR5448187) Gwiazda_D10_1 (SRR5448188) Gwiazda_D10_2 (SRR5448189, SRR5448190) Gwiazda_D10_3 (SRR5448191)	Leaves were collected after 6 DAS and 10 DAS
Orcheski et al. (2016)	*Malus X domestica*	4	Seedling leaves	WR1 (SRR3160181) WR2 (SRR3160208)	PR1 (SRR3160081) PR2 (SRR3160180)	Seedling leaves were harvested after 14 days
Liu et al. (2017)	*Solanum lycopersicum* *cv. M82*	4	Seedling leaves	SCK (SRR5282480) TCK (SRR5282476)	SD (SRR5282481) TD (SRR5282478)	The third seedling leaves were harvested at 10 DAS (Days after stress)
Salman et al. (2016)	*Vitis vinifera* *cv. Summer Black*	2	Mature leaves	Control (SRR3466603)	Treated (SRR3466604)	Mature leaves were collected with the interval of 5 days from 0 to 20 days
Liu et al. (2015)	*Triticum aestivum* *cv. TAM107*	6	Mature leaves	Control1 (SRR1542404) Control2 (SRR1542405)	Treated1 (SRR1542406) Treated2 (SRR1542407) Treated3 (SRR1542408) Treated 4 (SRR1542409)	Leaves were collected at 6 h after stress

### Read alignment, gene differential expression and annotation

For all the 9 articles, the latest available version of the corresponding crop genome and its annotation file were downloaded from Phytozome (https://phytozome.jgi.doe.gov). The raw data files were downloaded from NCBI SRA (https://www.ncbi.nlm.nih.gov/sra) and EMBL ArrayExpress (https://www.ebi.ac.uk/arrayexpress/) according to the accession number given in the article and converted to FASTQ format using SRA toolkit version 2.3.5. Raw data underwent pre-processing by trimming low-quality bases followed by adaptor sequence removal to obtain high-quality clean reads using cutadapt version 1.8.1. The pre-processed high-quality reads were mapped to the corresponding genome with HISAT2 version 2.1.0 [65] using the default parameters. The resulted output of HISAT2 was then used for the identification of differentially expressed genes using Cuffdiff tool in Cufflinks version 2.2.1 pipeline with default parameters. Up- and down-regulated genes with *p*-value < 0.05 were considered for downstream functional analysis. The DEGs selected were annotated using corresponding crop genome mapping file downloaded from the Phytozome. Custom made in-house Perl script was used for the selection of genes and mapping.

### Statistical and cluster analysis

The DEGs corresponding to each study separately analyzed, with p-value < 0.05, were then taken for the statistical analysis. Using p.adjust function of R, all the statistical tests were corrected for multiple comparisons using the Benjamini-Hochberg false discovery rate [66]. This approach can make the FDR at the desired level of α (in this study 0.05) by adjusting the *P*-values. R software was used for the statistical analysis. Differences among the selected studies were adjusted using the sample normalization. In order to remove systematic variation between different species, the normalization procedure served as a crucial pre-processing step to adjust for the different sample sequencing depths and other confounding technical effects. We used the geometric normalization method where FPKMs and fragment counts are scaled via the median of the geometric means of fragment counts across all libraries, as described in [67]. The dendrogram was generated for identifying the clustering patterns of the considered studies. The grouping of the clusters for dendrogram was done using the Euclidean distance measure.

### Gene set enrichment analysis

We mapped the entire differentially regulated gene IDs of each plant species to *Arabidopsis thaliana* and found out the corresponding best hit TAIR ID using the annotation file downloaded from Phytozome. We used MapMan [68] with the *Arabidopsis thaliana* mapping file (http://mapman.gabipd.org/) to map and visualize the metabolic overview, hormone regulation, secondary metabolism, transcription factors, and protein targeting. Firstly, we visualized the drought-regulated genes in common in at least 6 of 9 studies. Secondly, we visualized the drought-regulated genes in common between the three studies in seedlings; and finally in common between the five in mature leaves. The PageMan [69] analysis, plugin of MapMan, was used to visualize differences among metabolic pathways using Wilcoxon tests, no correction, and an over-representation analysis (ORA) cutoff value of 3. All the homologous TAIR IDs of the 9 studies were searched against the Database for Annotation, Visualization and Integrated Discovery (DAVID) version 6.8 [70] Web server (https://david.ncifcrf.gov/). The gene ontology information related to the biological process was extracted from the DAVID result.

### Gene mapping in crop chromosomes

The drought-regulated genes involved in abiotic stress, hormone metabolism and transcription factors were selected for the chromosome mapping. We found out the corresponding chromosome number, start and end of the drought-regulated gene IDs from the annotation file downloaded from Phytozome (https://phytozome.jgi.doe.gov) using custom made Perl script. These genes were then mapped towards the chromosome according to their chromosome number, start and end points.

### Protein-protein interaction network

NetworkAnalyst [71], a web-based tool for network-based visual analytics of protein-protein interaction networks, was used. The list of homologous TAIR IDs from 6 of 9 studies were uploaded and mapped against the STRING interactome database with default parameters (confident score cutoff = 900 and with experimental evidence) provided in NetworkAnalyst. The networks between drought-regulated genes in seedlings and in mature leaves corresponding to the list of the visualized genes in MapMan were also obtained. To study the key connectives and to simplify the large network, we selected “Minimum Network”.

## Additional files

Additional file 1: **Table S1.** The table consists of gene_ID, corresponding arabidopsis orthologs and log2 fold change values for significantly regulated genes (FDR < 0.05). The analysis results of each selected study has been given in seperate sheet along with the crop names. (XLSX 4134 kb)

Additional file 2: **Figure S1.** Clustering heatmap showing the hierarchical relationship among the studies selected for the analysis. Resulted log2FC values of the analysis for generating the tree was indicated. (TIF 163 kb)

Additional file 3: **Figure S2.** MapMan overview showing transcriptomic effects of drought in key categories selected such as secondary metabolism, cellular responses and signaling. Genes were identified as Arabidopsis orthologs of each genes of the analyzed plant species. Red means up-regulated and green means down-regulated. (TIF 226 kb)

Additional file 4: **Figure S3.** Protein-protein interaction network analysis predicted for genes commonly regulated in three seedling leaf studies performed in *Arabidopsis thaliana*, *Malus X domestica* and *Solanum lycopersicum* based on Arabidopsis knowledgebase. (TIF 332 kb)

Additional file 5: **Figure S4.** Protein-protein interaction network analysis predicted for genes commonly regulated in five mature leaf studies performed in *Vitis vinifera, Solanum tuberosum, Triticum aestivum, Zea mays* (study1) and *Zea mays* (study 2)based on Arabidopsis knowledgebase. (TIF 185 kb)

Additional file 6: **Figure S5.** Key genes encoding transcription factors, hormone metabolism and abiotic stress responses obtained from DAVID software were mapped in the respective chromosomes of the 7 crops. (TIF 536 kb)

Additional file 7: **Table S2.** Genes mapped in the chromosome which are involved in transcription factors, abiotic stress response and hormone metabolism. Arabidopsis ortholog, description and corresponding gene IDs were indicated. (XLSX 17 kb)

Additional file 8: **Figure S6.** The 27 key genes that were drought-regulated in at least 7 of 9 studies were mapped in the respective chromosomes of the 7 crops. (TIF 536 kb)

Additional file 9: **Table S3.** Genes mapped in the chromosome which are involved in atleast 7 of 9 studies. Arabidopsis ortholog, description and corresponding gene IDs were indicated. (XLSX 12 kb)

Additional file 10: **Table S4.** Genes involved in the leaf developmental stage that are commonly drought-regulated were shown. Common in seedling studies and common in mature studies were given in separate sheets. GeneID, description, expression pattern and binname were indicated. (XLSX 14 kb)

Additional file 11: **Table S6**. Genes involved in drought response corresponding to each of the study were shown. TAIR ID, description and log2FC were indicated. (XLSX 321 kb)

Additional file 12: **Table S5**. Genes involved in drought response at different leaf developmental stages (unique for seedling, unique for mature and common for seedling and mature) were shown. TAIR ID, log2FC and expression pattern were indicated. (XLSX 56 kb)

## Abbreviations

DAVID: Database for Annotation, Visualization and Integrated Discovery
DEGs: Differentially Expressed Genes
GO: Gene Ontology
ORA: Over-representation Analysis
TF: Transcription Factor
