# Supplementary figures and images for: Identification of key genes and its chromosome regions linked to drought responses in leaves across different crops through meta-analysis of RNA-Seq data
Source: BMC Plant Biol. 2019 May 10;19:194. doi: 10.1186/s12870-019-1794-y (PMC6511156; doi:10.1186/s12870-019-1794-y)

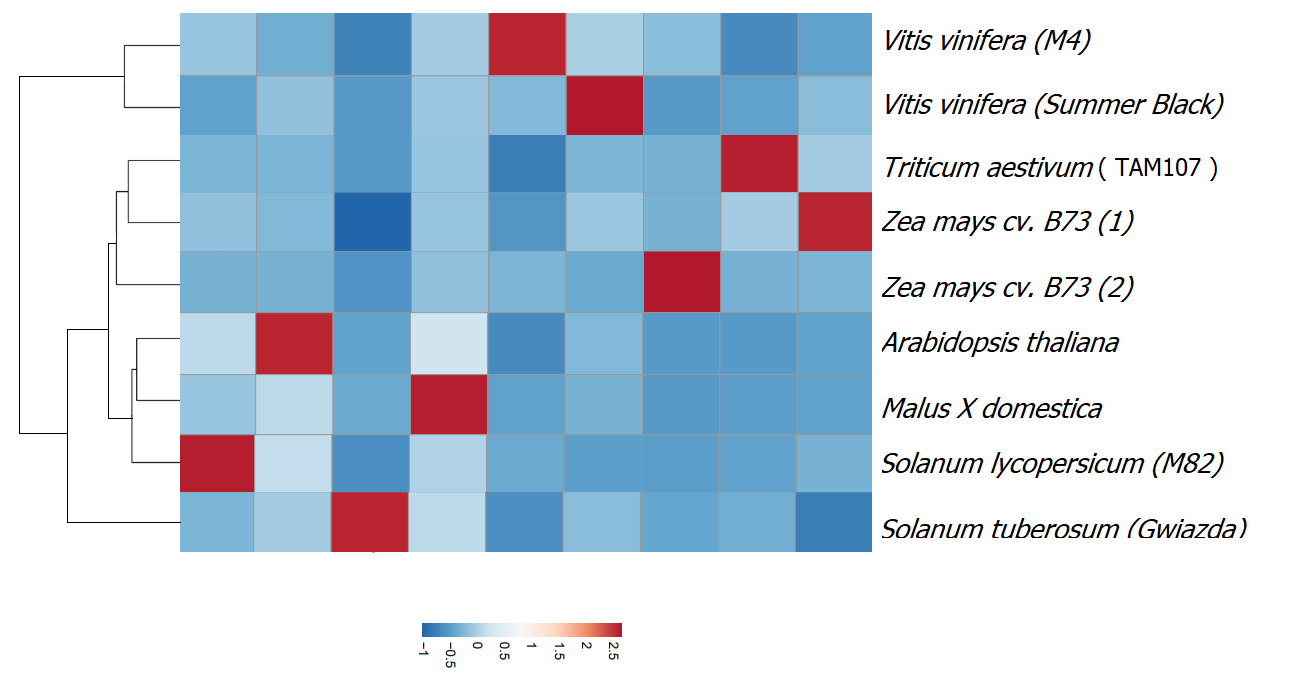

Supplement: Supplementary file 2 — Figure S1. Clustering heatmap showing the hierarchical relationship among the studies selected for the analysis. Resulted log2FC values of the analysis for generating the tree was indicated. (TIF 163 kb) [file 12870_2019_1794_MOESM2_ESM.tif]

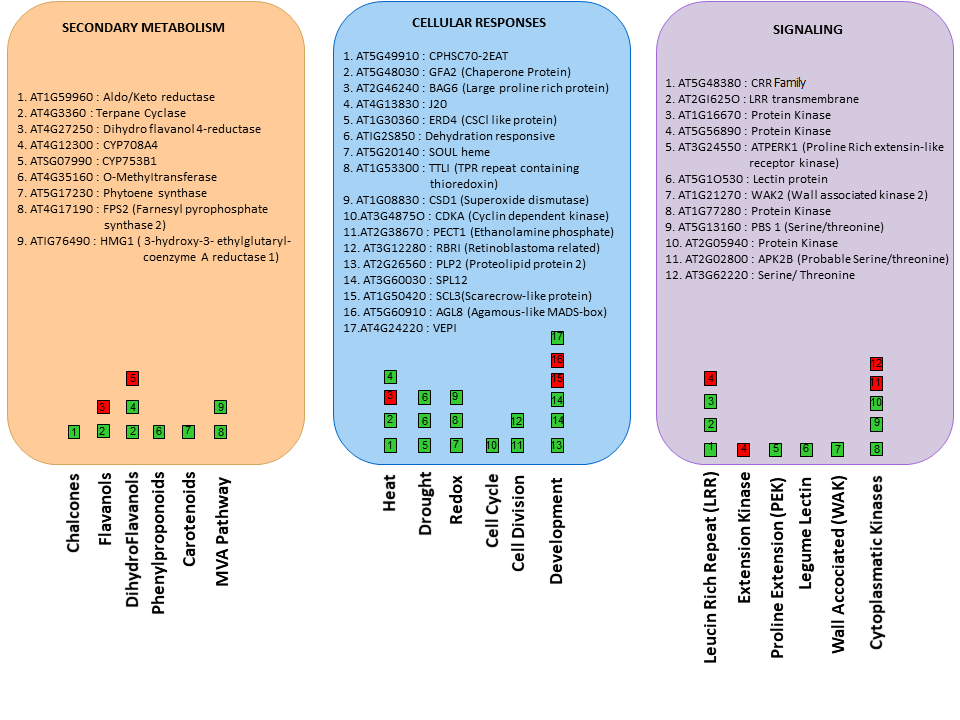

Supplement: Supplementary file 3 — Figure S2. MapMan overview showing transcriptomic effects of drought in key categories selected such as secondary metabolism, cellular responses and signaling. Genes were identified as Arabidopsis orthologs of each genes of the analyzed plant species. Red means up-regulated and green means down-regulated. (TIF 226 kb) [file 12870_2019_1794_MOESM3_ESM.tif]

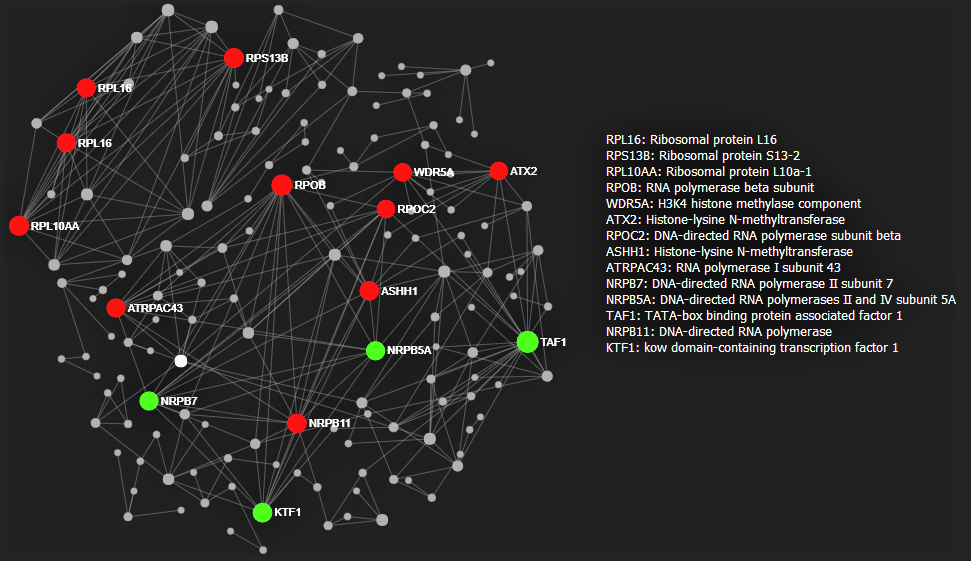

Supplement: Supplementary file 4 — Figure S3. Protein-protein interaction network analysis predicted for genes commonly regulated in three seedling leaf studies performed in Arabidopsis thaliana, Malus X domestica and Solanum lycopersicum based on Arabidopsis knowledgebase. (TIF 332 kb) [file 12870_2019_1794_MOESM4_ESM.tif]

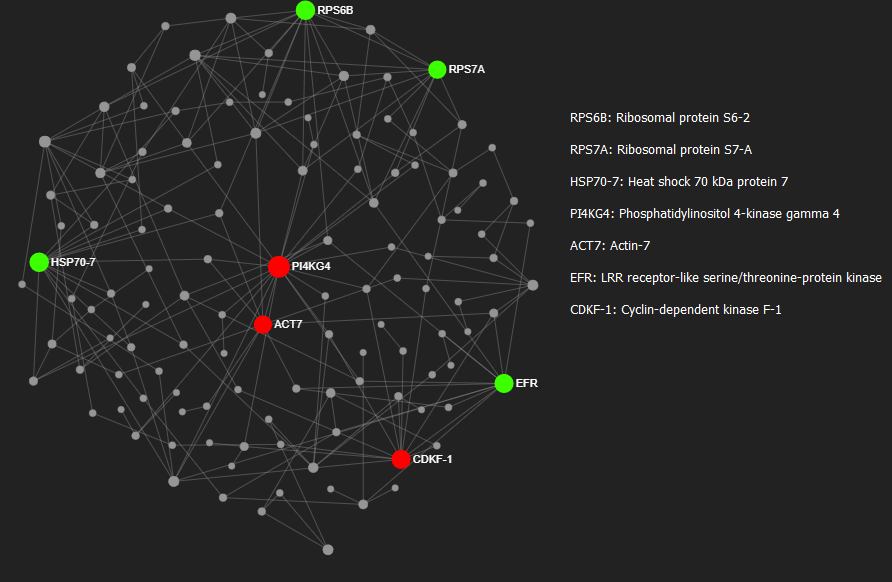

Supplement: Supplementary file 5 — Figure S4. Protein-protein interaction network analysis predicted for genes commonly regulated in five mature leaf studies performed in Vitis vinifera, Solanum tuberosum, Triticum aestivum, Zea mays (study1) and Zea mays (study 2)based on Arabidopsis knowledgebase. (TIF 185 kb) [file 12870_2019_1794_MOESM5_ESM.tif]

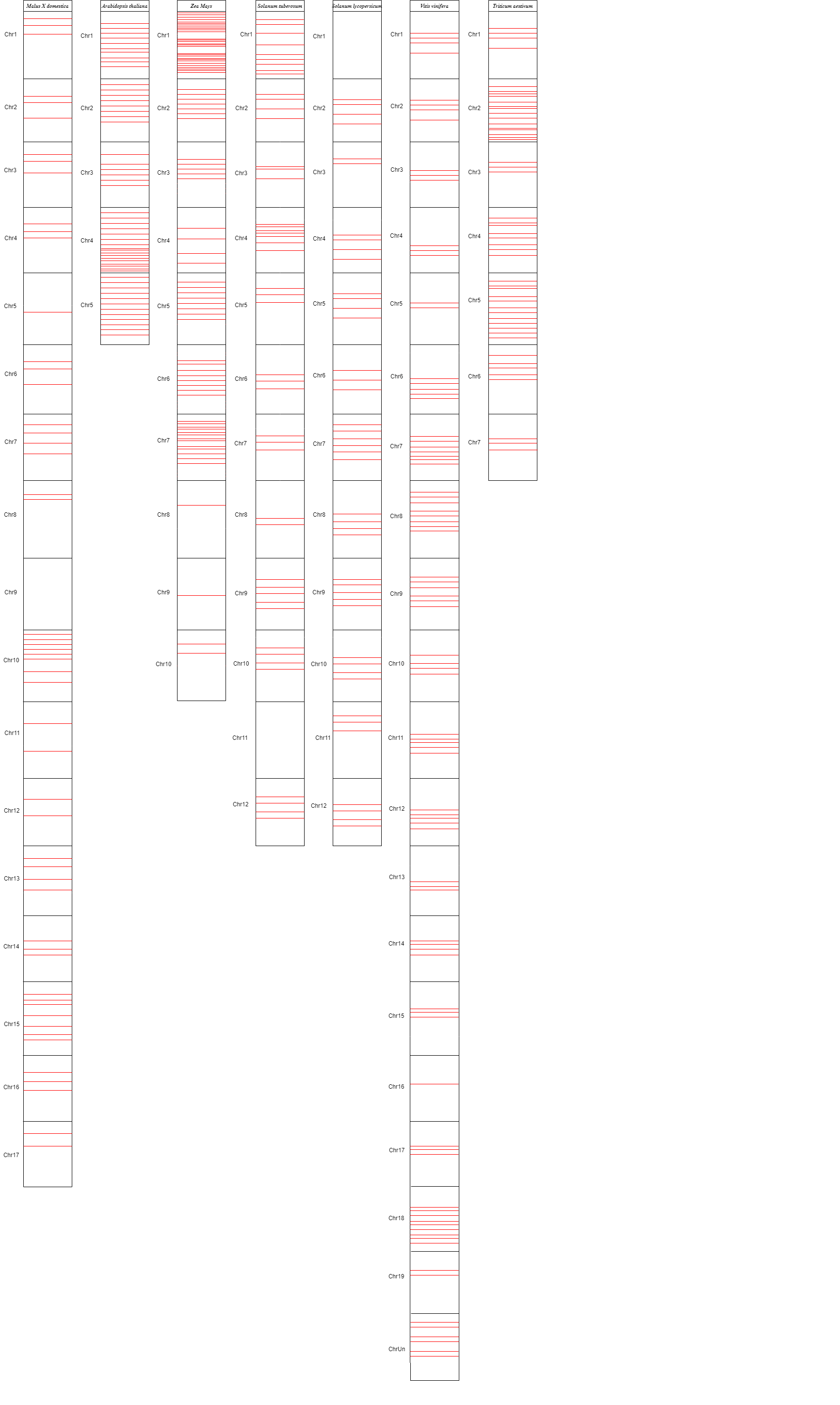

Supplement: Supplementary file 6 — Figure S5. Key genes encoding transcription factors, hormone metabolism and abiotic stress responses obtained from DAVID software were mapped in the respective chromosomes of the 7 crops. (TIF 536 kb) [file 12870_2019_1794_MOESM6_ESM.tif]

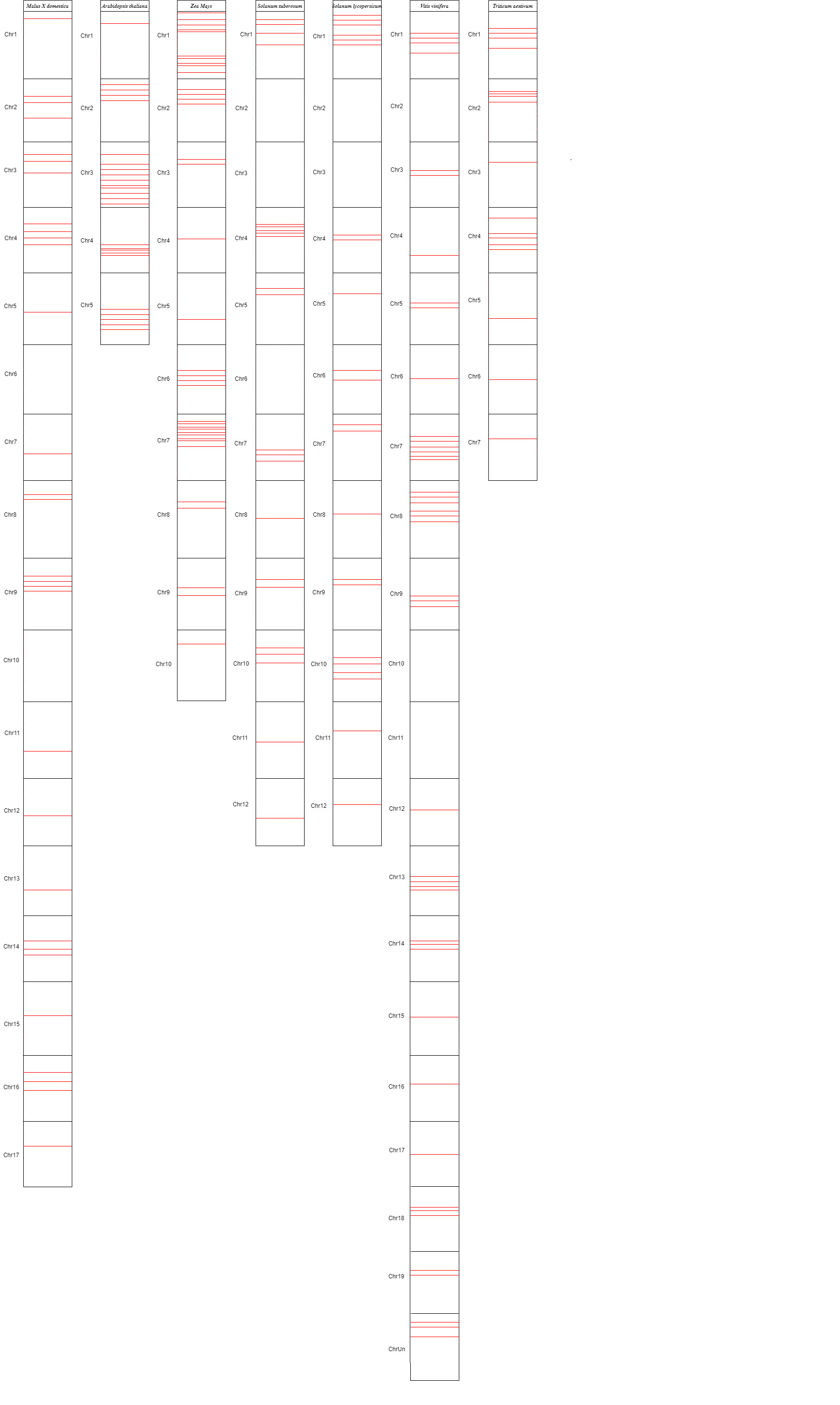

Supplement: Supplementary file 8 — Figure S6. The 27 key genes that were drought-regulated in at least 7 of 9 studies were mapped in the respective chromosomes of the 7 crops. (TIF 536 kb) [file 12870_2019_1794_MOESM8_ESM.tif]
